# Injury From Nematode Lung Migration Induces an IL‐13‐Dependent Hyaluronan Matrix

**DOI:** 10.1002/pgr2.70012

**Published:** 2024-11-25

**Authors:** Rebecca J. Dodd, Dora Moffatt, Monika Vachiteva, James E. Parkinson, Brian H. K. Chan, Anthony J. Day, Judith E. Allen, Tara E. Sutherland

**Affiliations:** ^1^ Wellcome Centre for Cell Matrix Research, School of Biological Sciences, Faculty of Biology, Medicine & Health University of Manchester Manchester UK; ^2^ Lydia Becker Institute of Immunology and Inflammation, School of Biological Sciences University of Manchester Manchester UK; ^3^ Division of Infection, Immunity & Respiratory Medicine, School of Biological Sciences, Faculty of Biology, Medicine & Health University of Manchester Manchester UK; ^4^ Division of Cell Matrix Biology & Regenerative Medicine, School of Biological Sciences, Faculty of Biology, Medicine & Health University of Manchester Manchester UK; ^5^ Institute of Medical Sciences University of Aberdeen Aberdeen UK

**Keywords:** HC·HA complexes, hyaluronan, IL‐13, lung damage, tissue repair, Tsg6, type 2 immunity

## Abstract

A consistent feature of lung injury is a rapid and sustained accumulation of hyaluronan (HA). The rodent gut‐dwelling nematode *Nippostrongylus brasiliensis* (Nb) induces tissue damage as it migrates through the lungs. Type 2 immune responses are essential for the repair of the lungs, hence Nb infection is a well‐established model to study immune‐mediated lung repair. We found that Nb infection was associated with increased HA in the lung, which peaked at d7 post‐infection (p.i.). Deposition of HA in the alveolar epithelium correlated with regions of damaged tissue and the type 2 immune response, which is characterized by eosinophilia and increased type 2 cytokines such as IL‐13. Consistent with the accumulation of HA, we observed increased expression of the major synthase *Has2*, alongside decreased expression of *Hyal1, Hyal2*, and *Tmem2*, which can degrade existing HA. Expression of Tsg6 was also increased and correlated with the presence of inter‐α‐inhibitor heavy chain–HA complexes (HC·HA) at d7 p.i. Using IL‐13‐deficient mice, we found that the accumulation of HA during Nb infection was IL‐13 dependent. Our data thus provide further evidence that IL‐13 is a modulator of the HA matrix during lung challenge and links IL‐13‐mediated HA regulation to tissue repair pathways.

## 1 Introduction

Accumulation of the extracellular matrix (ECM) component hyaluronan (HA) is a consistent feature following lung injury and inflammation. Increases in this polysaccharide have been described not only in animal models of viral infection [1, 2, 3, 4], allergic pathology [5, 6, 7], and pollutant/chemical stimulation [8, 9] but also in human lung disease [4, 10, 11, 12, 13, 14]. Therefore, an increase in HA appears to be a highly conserved response to pulmonary challenge.

Inflammatory mediators, such as lipopolysaccharide (LPS) [15], and the pro‐inflammatory cytokines, TNF‐α and IL‐1β (via NFκB), have been reported to induce HA [16, 17, 18]. In addition, the anti‐inflammatory cytokine IL‐10 can regulate HA production during wound repair, for example, as part of scar‐free fetal healing [19, 20, 21, 22]. In recent work, we demonstrated that the type 2 cytokine IL‐13 is also an inducer of HA in the lung during SARS‐CoV2 infection in mice [4]. IL‐13 neutralization in infected mice led to reduced levels of HA in the alveolar epithelium and downregulated HA synthases in the lung. Furthermore, direct delivery of recombinant IL‐13 in vivo was able to drive HA accumulation in the lungs of naïve mice [4]. This finding is surprising given that IL‐13 is more often associated with parasite and allergic responses than the response to viral challenges.

In addition to its accumulation during injury and inflammation, HA becomes covalently modified with so‐called heavy chains (HC) from the serum proteoglycan, inter‐alpha‐inhibitor (IαI) [23, 24]. Formation of HC·HA occurs in a two‐step transesterification process catalyzed by the anti‐inflammatory protein tumor necrosis factor‐stimulated gene 6 (TSG‐6 or Tsg6 in mice) [23, 25]. HC·HA matrices have been observed in response to allergic asthma, viral infection, and bleomycin challenge [1, 2, 14, 26]. In all cases, the detection of such matrices coincides with the accumulation of HA and immune infiltration into the lung. Tang et al. [2] recently found that synthesis of IαI, required for HC·HA matrix formation, was induced locally in the lung during influenza infection rather than being made in the liver, which is the usual source of IαI for HC transfer [2, 27, 28]. While the presence of such matrices has been well‐defined and frequently described in ovulation [25] and gut development [29], their functions during lung health, injury, and repair remain far less clear [30].

Infection of mice with the helminthic parasite *Nippostrongylus brasiliensis* (Nb) is a well‐established model to study immune‐mediated lung injury and repair [31, 32, 33, 34, 35]. Larvae infect by penetrating the skin before rapidly migrating to the lungs (by Day 1 p.i.), where they burst through capillary beds into the lung parenchyma [36]. Entry into the lung is accompanied by host neutrophilic responses which contribute to tissue damage and bleeding [32]. Parasites are then coughed up and swallowed entering the gut (where they mature and reproduce) before being expelled. Soon after the parasite has left the lungs, a strong type 2 immune response, characterized by cytokines including IL‐4, IL‐5, IL‐9, IL‐10, and IL‐13, is initiated in the lungs [37]. This type 2 immune response is essential for initiating the repair of the damaged tissue [32, 38, 39]. Effective tissue repair requires precise remodeling of ECM components including collagens, glycosaminoglycans, and proteoglycans. These remodeling events must be appropriately controlled; otherwise, scarring and fibrosis can ensue, which ultimately impede tissue function [40, 41, 42, 43].

Here, we used the mouse model of Nb lung infection and found that accumulation of HA correlates with the peak of the type 2 immune response induced by the infection. The HA matrix formed during infection included the presence of covalently attached HCs and appeared to resolve after the peak of inflammation. We show that the formation of this matrix is dependent on IL‐13, providing further evidence that IL‐13 is a modulator of HA in response to lung challenges. The Nb infection model in mice represents an excellent tool to study immune drivers of HA matrix formation after lung injury and help define the role of such matrices in lung health and repair.

## 2 Materials and Methods

### 2.1 Experimental Animals

All experiments involving animals were performed in accordance with the UK Animals Act (1986) under a Project License granted by the UK Home Office and approved by the University of Manchester Animal Welfare and Ethical Review body (PP4115856). Wild‐type C57BL/6J^crl^ mice were obtained from Charles River (Margate, UK). IL13eGFP^*+/‐*^ (HET) and IL‐13eGFP^*‐/‐*^ (KO) mice (on a C57BL/6 J background; originally from Professor Andrew McKenzie [44]) were bred at the University of Manchester with wild‐type (IL13eGFP^*+/+*^, WT) littermates used as controls. Experimental mice were mixed sex and between 10 and 16 weeks old at the time of infection (indicated in individual figure legends) with groups age‐ and sex‐matched where possible. All mice were housed in individually ventilated cages with between 3 and 6 mice per cage and maintained in facilities at the University of Manchester.

For Nb infection, the parasite was maintained as described [45]. Cages were randomly assigned to treatment groups with infected animals maintained in separate cages from uninfected animals. Mice were infected with 250 third‐stage larvae (L3) on Day 0 (d0) via sub‐cutaneous injection into the scruff (in 200 μL PBS). Mice were euthanized at various timepoints (by either overdose with anesthetic or asphyxiation in a rising concentration of carbon dioxide) post‐infection (p.i.) as indicated in figures and legends.

### 2.2 Isolation of Lung and Bronchoalveolar Lavage (BAL) Cells for Flow Cytometry Analysis

**Figure 1:**
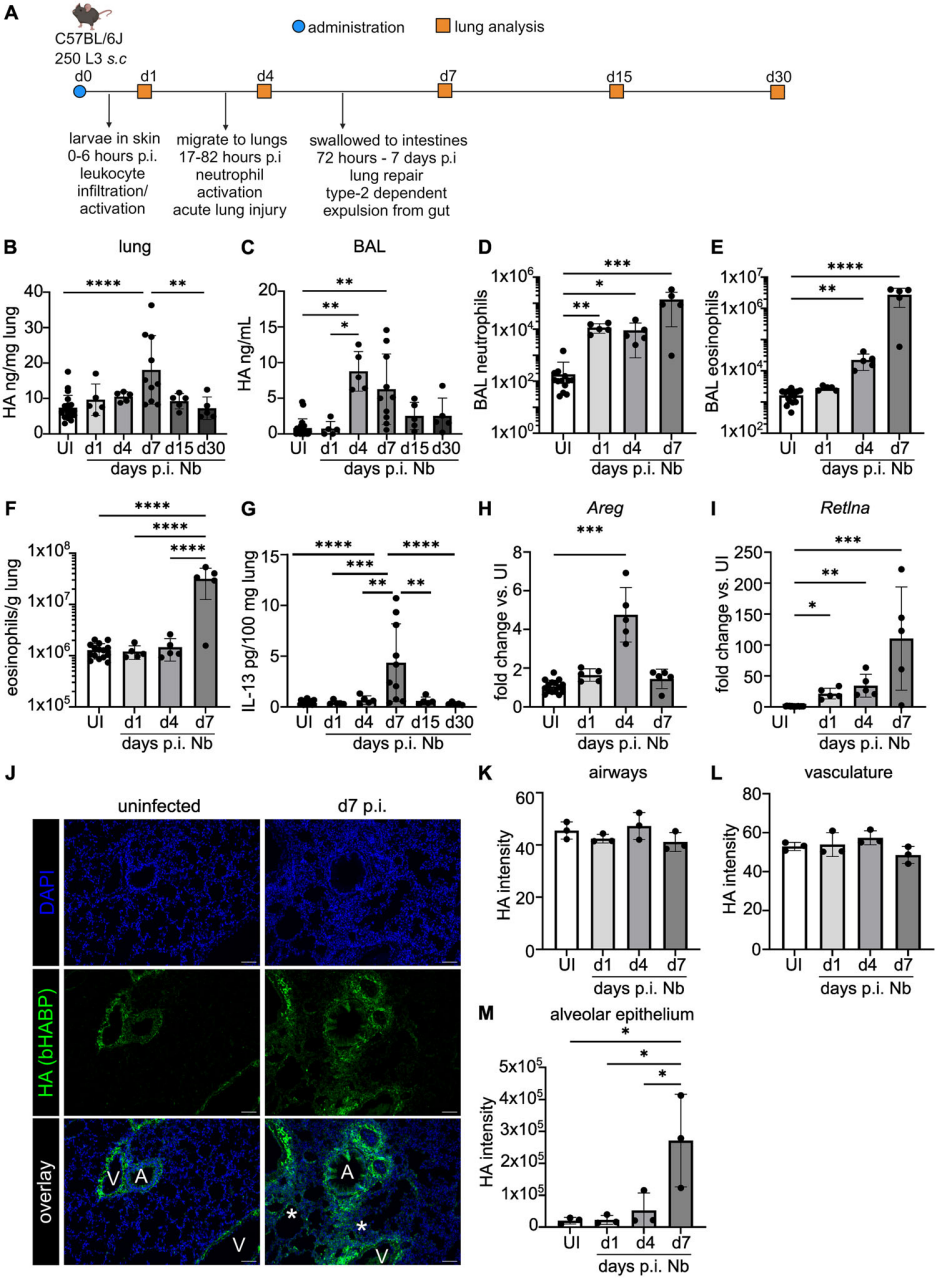
Nb infection increases HA in the lungs. (A) Schematic showing timepoints of lung analysis following Nb infection in C57BL/6 wild‐type mice and key timepoints in parasite migration post‐infection (p.i.). HA levels, measured by ELISA, in (B) lung homogenate (normalized to lung weight) and (C) bronchoalveolar lavage fluid (BALF) from wild‐type uninfected (UI) or Nb‐infected female C57BL/6 J mice, as per (A). Total number of immune cells measured by flow cytometry: (D) BAL neutrophils, (E) BAL eosinophils, and (F) lung eosinophils. Eosinophil numbers in the lung were normalized to lung weight. (G) IL‐13 levels in lung homogenate measured by Legendplex ELISA, normalized to lung weight. Gene expression of (H) *Areg* and (I) *Retnla* in whole lung measured by qPCR. Values are reported as fold change compared to UI controls with all values normalized to expression of housekeeping genes (*Rn18s* and *Rpl13a*.) (J) Immunofluorescence staining of lung tissues to visualize HA (bHABP, green) and cell nuclei (stained with 4′,6‐diamidino‐2‐phenylindole [DAPI], blue) in UI and Nb mice at d7 p.i. White letters indicate regions of the lung; airways (A), vessels (V) or alveolar epithelium (*). Images are representative of 3 mice per group. Scale bar = 100 μm. HA staining intensity was quantified in (K) airways, (L) vasculature, and (M) alveolar epithelium. In (B−M), datapoints depict individual animals and are shown as the mean ± standard deviation (SD). Data in (B−M) are pooled from two‐different experiments (with 10‐week‐old female mice) and were analyzed by Analysis of Variance (ANOVA) with Tukey's multiple comparison test. *p < 0.05, **p < 0.01, ***p < 0.001, ****p < 0.0001.

To collect BAL cells, the trachea was cannulated, and the airways were washed with 0.4 mL PBS four times. Cells were collected by centrifugation (400*g*) and BAL fluid (BALF; supernatant) stored at −80°C for further analysis by ELISA. Lung cells were collected from the inferior and post‐caval lobes. Tissue was minced with blunt nose scissors and then incubated for 45 min in a 37°C shaking incubator in 1 mL of RPMI containing 0.4 U/mL Liberase TL (Sigma) and 80 U/mL DNase Type I (Invitrogen). Digestion was stopped with ice‐cold RPMI with 10% (v/v) fetal bovine serum (FBS), and cells were filtered through 70 μm cell strainers. Red blood cells were lysed using ACK Lysis Buffer (Invitrogen) before cells were counted and viability assessed (using a Cellometer Automated cell counter, Nexcelom Bioscience). For staining, equal numbers of lung or BAL cells were washed in PBS and stained for 10 min at room temperature (RT) with Live Dead Aqua (Invitrogen) followed by incubation with Fc block (5 μg/mL CD16/CD32 in PBS with 2% FBS and 2 mM EDTA [FACS buffer]) for 15 min at 4°C. Surface staining was performed for 30 min at 4°C in FACS buffer (see Supporting Information S1: Table 1 for antibodies and fluorophores) and then cells were fixed for 20 min at RT (IC fixation buffer, Invitrogen) and stored at 4°C until acquisition. All cells were then washed and resuspended in FACS buffer and acquisition performed using a BD LSRFortessa instrument running BD FACSDiva software (BD Biosciences). Analysis of flow cytometry data was performed using FlowJo (version 10.10; BD Biosciences), and cells were identified according to the gating strategy shown in Supporting Information S2: Figure 1.

### 2.3 ELISA

The superior lung lobe was flash‐frozen on dry ice at the point of collection and then homogenized in PBS using the Tissue Lyser II system (Qiagen). Homogenates were centrifuged at 16,000*g* for 5 min and supernatants were stored at −80°C before analysis. HA quantification was performed using HA DuoSet ELISA (R&D Systems) according to the manufacturer's instructions. Lung homogenate was diluted at 1:80 and BALF was diluted at 1:10. Cytokine levels were determined using a Luminex mouse Th cytokine panel kit (BioLegend) according to the manufacturer's instructions and data were acquired using a BD Fortessa flow cytometer (BD Biosciences). All concentrations were normalized to weight of the tissue lobe.

### 2.4 qPCR

The middle lung lobe was stored in RNALater solution (Invitrogen) at −80°C before RNA was extracted using PureLink RNA Mini Kit (Invitrogen) following the manufacturer's instructions. RNA yield was determined using a Nanodrop. RNA (500 ng) was reverse‐transcribed using 50 U Tetro reverse transcriptase (Bioline), 40 mM dNTPs (Promega), 0.5 µg cDNA poly A synthesis primer (Jena Bioscience), and recombinant RNasin inhibitor. cDNA was stored at −20°C until qPCR analysis. Reverse transcription qPCR was performed on a Lightcycler 480 II (Roche) using Brilliant III SYBR Green master mix and specific primer pairs (Integrated DNA Technologies) for genes of interests (see Supporting Information S1: Table 2). Amplification was analyzed using the second derivative maximum algorithm (Roche) and expression of genes of interest were normalized to expression of housekeeping genes (*Rn18s* and *Rpl13a*) and the control group (uninfected) using the ddCT method [46].

### 2.5 Tissue Histology and Immunofluorescence

The left lung lobe was inflated (via its cannulated trachea) with 10% (v/v) neutral buffered formalin (NBF) and fixed overnight before storage in 70% (v/v) ethanol. Tissues were processed through an alcohol series and embedded in paraffin wax. Sections of 5 μm were cut using a microtome and mounted onto slides (Superfrost Plus Adhesion). H&E staining was performed using standard protocols and sections were imaged with a 3D Histech Pannoramic P250 slide scanner. Mean linear intercept analysis (to assess lung epithelium damage) was performed as described in [47] using ImageJ (FIJI, v1.53q). Ten non‐overlapping, randomly chosen regions of interest (ROIs, devoid of airways or vasculatures) per mouse were analyzed and the average score was calculated.

For immunofluorescence staining, slides were dewaxed through xylene and rehydrated through an ethanol series before heat‐mediated antigen retrieval using 10 mM Tris, 1 mM EDTA, pH 9.0 + 0.05% (v/v) Tween‐20 (T‐20) buffer. Slides were heated in buffer using a microwave for 20 min. After antigen retrieval, slides were cooled and then tissues permeabilized in 0.5% (v/v) Triton‐X100 (in PBS) buffer for 20 min at RT. Slides were then incubated in blocking buffer (10% [v/v] donkey serum, 1% [w/v] BSA, 0.05% [v/v] T‐20 in PBS) for 1 h at RT and then endogenous biotin blocked using Avidin‐Biotin blocking kit (Invitrogen). Slides were incubated with primary antibodies (Supporting Information S1: Table 3) diluted in blocking buffer overnight at 4°C. The following day, slides were washed (2 × 5 min) in PBS with 0.05% (v/v) T‐20 followed by PBS and then incubated with secondary antibodies (Supporting Information S1: Table 3) for 1 h at RT and then washed as before. DAPI (1:40,000 in PBS) was used to stain nuclei and cover slips were applied using a fluorescence anti‐fade mounting medium. Imaging was performed using a 3D Histech Pannoramic P250 slide scanner or EVOS FL scope with the experimenter masked to the mouse identity. Image analysis was performed using ImageJ (FIJI, v1.53q). Background autofluorescence (determined from sections stained with secondary antibody only) was subtracted from images. Positive pixel intensity and area were measured on ROIs (airways, vessels, or parenchyma) drawn on images for which positive stain threshold had been applied. Staining intensity was normalized to area or length of basement membrane (airways and vessels, respectively) and averaged across five ROIs per section per mouse.

### 2.6 Western Blot Analysis

Lung homogenate (see ELISA) was treated with 2.5 U *Streptomyces hyaluronlyticus nov. sp*. (EMD Millipore) for 2 h at 37°C in a water bath to fragment HA. An equal volume of homogenate was incubated 1:1 with water for the non‐enzyme‐treated control. All samples were mixed with x4 sample loading buffer (Invitrogen) containing 5% mercaptoethanol and boiled for 10 min at 75°C before loading onto 4%−12% Bis‐Tris polyacrylamide gels (Invitrogen). Samples were electrophoresed at 150 V for 1.5 h (or until the 22‐kDa marker of SeeBlue Plus 2 protein ladder (Invitrogen) had run to the bottom of the gel). HC·HA controls were generated essentially as described [48] but using normal mouse serum (Sigma) as the IαI source and recombinant‐mouse Tsg6 (R&DSystems). Proteins were transferred to nitrocellulose membranes (Amersham) at 35 V for 1.5 h in x1 BOLT transfer buffer (Invitrogen) containing 10% methanol. Membranes were blocked using 10% (w/v) skimmed milk powder (Sigma) in x1 PBS + 0.2% (v/v) T‐20 for 1 h and then incubated with primary antibody (1:5000, rabbit anti‐human polyclonal antibody against IαI [Dako], diluted in 5% [w/v] milk‐PBS‐T‐20) overnight at 4°C on a rocking platform. Membranes were washed in PBS + 0.2% (v/v) T‐20 before incubation with secondary antibody (donkey anti‐rabbit IgG 800Cw; Licor; 1:10,000) for 1 h at RT. Blots were imaged on a Licor Odyssey Clx imager (Licor Biosciences GmH).

### 2.7 Statistical Analyses

Statistical analyses were performed using GraphPad Prism (v10.2.3). Normality of data sets was assessed using Shapiro–Wilks tests. Where appropriate data sets were log‐transformed to meet normality requirements. Differences between timepoints and groups were then assessed using one‐way ANOVA with Tukey's multiple comparisons test for normally distributed data sets or Kruskal–Wallis tests with Dunn's comparison for non‐normal distributions. Comparisons with *p* < 0.05 were considered significant.

## 3 Results

### 3.1 Nb Infection Increases HA in the Lungs

To investigate whether Nb infection altered HA within the lungs, mice were infected with 250 L3 larvae and tissue harvested at various timepoints p.i. (Figure 1A). HA levels were measured in lung homogenate and found to be significantly increased (compared to naïve mice) at d7 p.i. (Figure 1B). However, by Day 15 p.i., the amount of HA had returned to levels found in naïve lung tissue. HA in BALF was also increased upon infection, with a peak increase at d4 p.i. and remaining significantly elevated at d7 but not the later timepoints (Figure 1C). Consistent with previous findings, the early immune response to Nb infection was characterized by elevated neutrophils in the airways at d1 p.i. (Figure 1D), corresponding to acute lung injury [32, 47, 49]. At later timepoints, the immune response was dominated by eosinophilia, observed both in the airways (BAL) (Figure 1E) and the lung (Figure 1F) at d7 p.i. Increased eosinophil numbers corresponded to a type 2 immune response, which is critical for tissue repair [32, 38], and was associated with a fivefold increase in IL‐13 concentration in the lungs (Figure 1G). Expression of the wound‐response gene *Areg* (Amphiregulin; Figure 1H) was induced at d4 p.i., consistent with previous studies [50]. *Retnla*, encoding Resistin‐like molecule alpha (RELMα; a type 2‐induced molecule), expression was also increased p.i., with a peak at d7 (Figure 1I). Both Amphiregulin and RELMα are understood to be critical in the host repair response to Nb‐induced damage [33, 35, 51].

We next wanted to determine whether HA was localized to specific regions in the lung tissue following infection. In the lungs of uninfected animals, HA localized to the sub‐epithelial layer of the airways (as part of the basement membrane) and was associated with the endothelium of the vasculature (“uninfected” left‐hand side panel in Figure 1J) [52]. With infection, no significant differences were observed in the intensity of HA staining around the airways (Figure 1J (right hand “d7 p.i.” panel) and K) or vasculature (Figure 1J,L) at any timepoint. However, HA staining was significantly increased in the alveolar epithelium of infected lungs at d7 p.i. compared to uninfected mice (Figure 1J,M). Quantification of HA staining by histology was consistent with the increased HA measured in lung homogenates at d7 p.i. with Nb (Figure 1B). Our findings that HA is deposited predominantly near the alveolar epithelium aligns with what is known about the areas of lung tissue damaged during Nb infection [31, 32, 53].

### 3.2 Infection With Nb Leads to Changes in Expression of HA‐Related Genes

**Figure 2:**
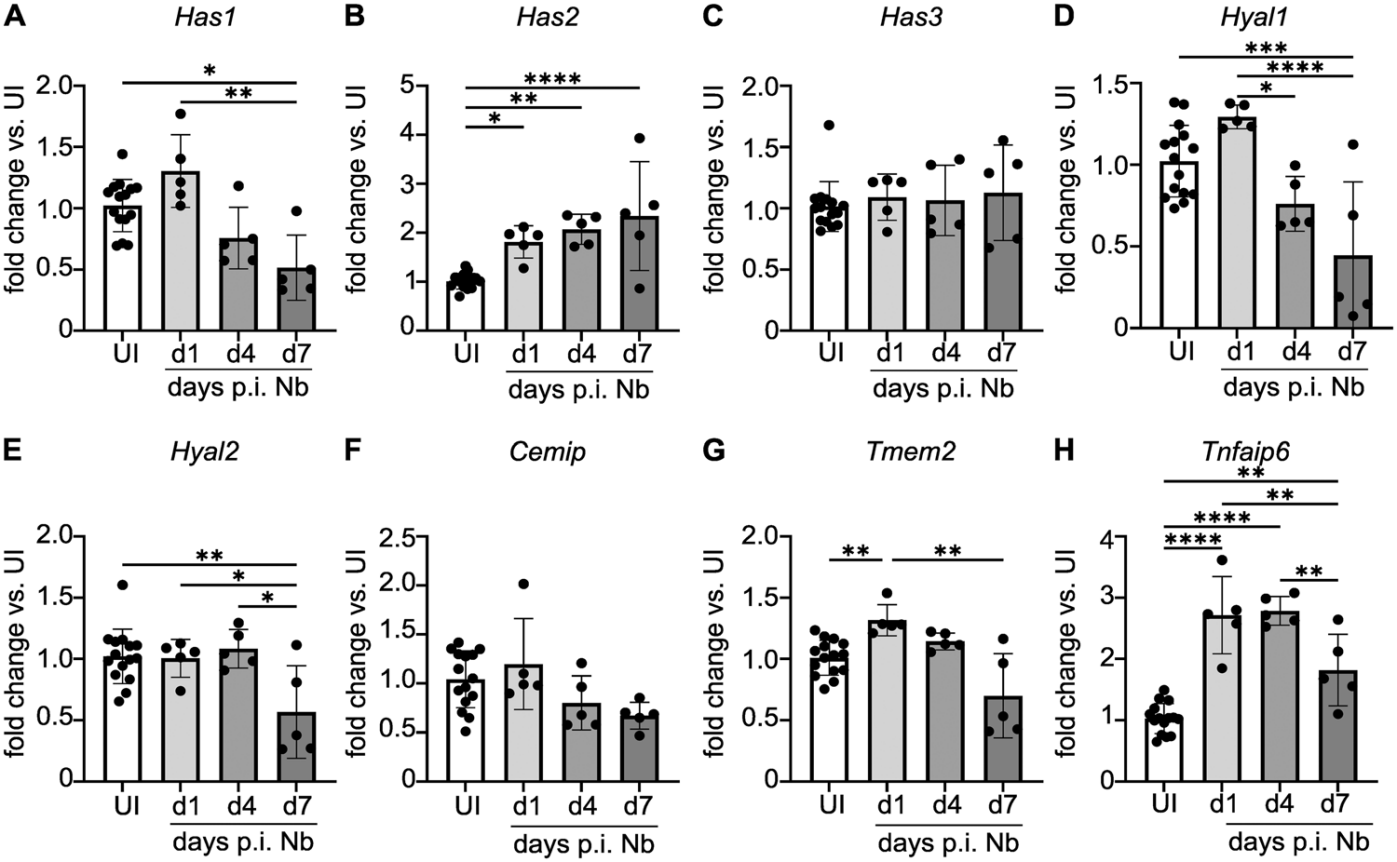
Nb infection is associated with changes in expression of HA‐related genes. mRNA expression of (A) *Has1*, (B) *Has2*, (C) *Has3*, (D) *Hyal1*, (E) *Hyal2*, (F) *Cemip*, (G) *Tmem2*, and (H) *Tnfaip6* (encoding Tsg6) measured in whole lung from C57BL/6 J female mice infected with Nb and harvested at d1, d4, or d7 p.i. Values are reported as the fold change compared to the uninfected (UI) controls for each timepoint. All values are normalized to expression of the housekeeping genes, *Rn18s* and *Rpl13a*. Datapoints depict individual animals and are shown as mean ± SD. Data are representative of two experiments. Differences between groups were analyzed by ANOVA with Tukey's multiple comparison test or ANOVA with Kruskal–Wallis test (for non‐normally distributed data). *p < 0.05, **p < 0.01, ***p < 0.001, ****p < 0.0001.

Having established that HA increased in the lungs following helminth infection, we sought to determine whether such changes corresponded to altered expression of genes associated with HA metabolism and modification. Unexpectedly, expression of the HA synthase gene, *Has1*, was significantly decreased in lung homogenate at d4 and d7 p.i. (Figure 2A). However, *Has2* was significantly increased (compared to uninfected mice) at d1, d4, and d7 p.i., with peak expression at d7 (Figure 2B) consistent with an increase in lung HA (Figure 1B,J). Expression of *Has3* was not altered in the lung between d1 and d7 p.i. (Figure 2C), highlighting that not all lung HA synthases were influenced in the same way in response to Nb infection. Expression of some hyaluronidases was also modulated in the lung p.i. The intracellular hyaluronidase *Hyal1* was significantly decreased at d7 p.i. compared to uninfected controls and reduced at d4 p.i. (compared to d1 p.i.; Figure 2D), while *Hyal2* expression was significantly decreased at d7 p.i. (Figure 2E). Expression of the cell‐surface hyaluronidase, *Cemip*, was reduced over time in response to infection, but this reduction was not significant (Figure 2F). *Tmem2* expression increased initially at d1 p.i. but significantly decreased at d7 p.i. (compared to d1 p.i.; Figure 2G). To assess whether HA may be present in its HC‐modified form in the lungs following Nb infection, expression of *Tnfaip6* (which encodes Tsg6) was measured and found to be significantly increased at d1, d4, and d7 p.i. (Figure 2H). Taken together, these results suggest that metabolism of HA is markedly altered in response to Nb infection.

### 3.3 Nb Infection Induces a HC·HA Matrix

**Figure 3:**
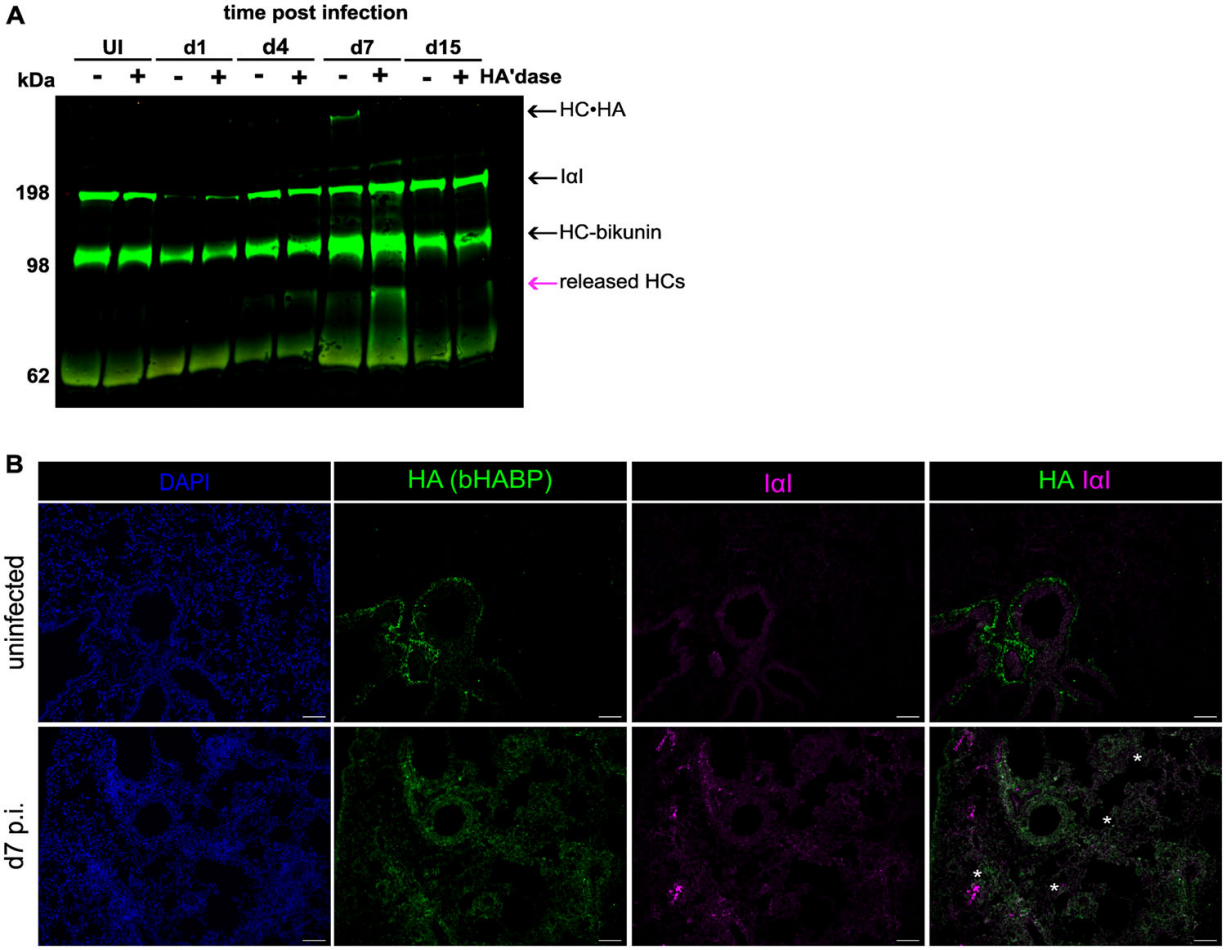
Nb infection is associated with the presence of a HC·HA matrix. Lung homogenate analyzed by western blot analysis with and without hyaluronidase treatment to release HCs from HC·HA matrices present in tissue. (A) Samples from d1, d4, d7, and d15 p.i. compared to an uninfected control (UI). Blots were probed with anti‐IαI antibody. Blots shown are representative of 3 repeats with samples from different mice across 2 independent experiments. (B) Staining of lung tissue with IαI (anti‐IαI; pink) and HA (bHABP, green) from uninfected mice at d7 p.i. Cell nuclei are stained with DAPI (blue). White asterisks (*) indicate areas of HA and IαI colocalization in areas of lung damage. Images are representative of *n* = 4 mice per group. Scale bar: 100 μm.

Given the increase in HA at d7 p.i. (Figure 1B,M) and increased expression of the gene encoding Tsg6 (Figure 2H), we asked whether a HC·HA matrix formed in the lungs during Nb infection. Lung homogenates collected from uninfected and Nb‐infected mice at various timepoints were treated with and without hyaluronidase and analyzed by Western blot analysis to identify released HC, indicative of a covalent HC·HA matrix. A band with an apparent molecular weight of 180 kDa corresponding to IαI was detectable in both uninfected and infected lung tissue samples across all timepoints (Figure 3A and Supporting Information S2: Figure 2). A band corresponding to released HCs was observed in hyaluronidase‐treated infected samples from some mice at d4 p.i. and in all mice at d7 p.i. (Figure 3A, Supporting Information S2: Figure 2). Released HCs were not detected in the homogenates of lungs from d15 or d30 p.i., suggesting that the covalent matrix was removed over time (Figure 3A, Supporting Information S2: Figure 2). Immunofluorescence staining of lung tissue sections at d7 p.i. showed colocalization of HA and IαI throughout the alveolar epithelium of infected mice only. In uninfected mice, this colocalization was not observed, consistent with no HC·HA matrices detected in uninfected mice (Figure 3A). Given that the alveolar epithelium is known to be damaged by the parasite (Figure 3B), this could suggest that the HC·HA matrix could be an important component of the tissue response to injury.

### 3.4 IL‐13 Is Needed for Full HA Matrix Formation After Nb Infection

**Figure 4:**
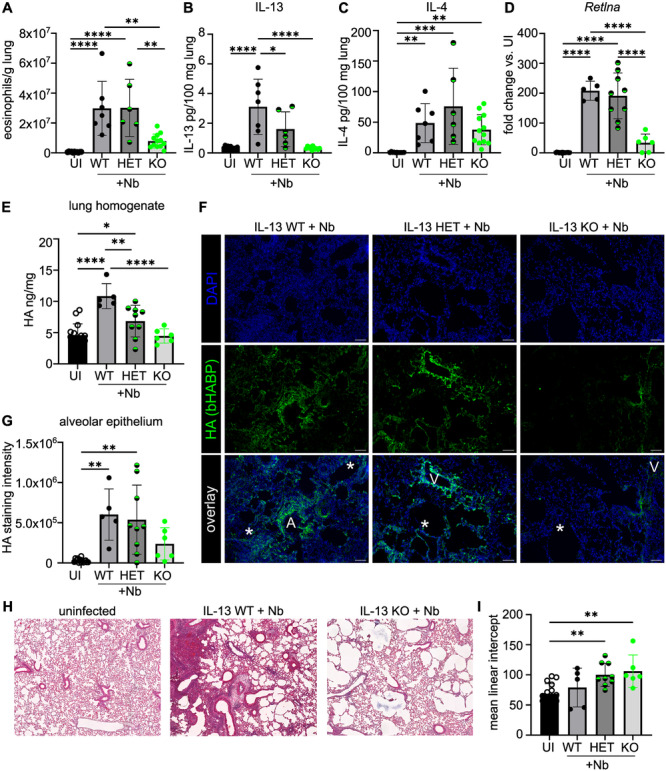
IL‐13‐deficient mice have less HA in the lung after Nb infection. IL‐13^*+/+*^ (WT), IL‐13^−*/+*^ (HET), and IL‐13^*−/−*^ (KO) mice (male and female, 11−16 weeks old) were infected with 250 L3 larvae and lung tissue harvested at d7 p.i. (A) Total number of eosinophils in lung tissue at d7 p.i. (normalized to lung weight) determined by flow cytometry. Quantification of type 2 cytokines (B) IL‐13 and (C) IL‐4 in lung homogenate at d7 p.i. by Legendplex ELISA. Amounts are normalized to weight of lung tissue. (D) Change in expression of *Retnla* in whole lung at d7 p.i. measured by qPCR. Values are reported as the fold change compared to UI mice and all values are normalized to expression of housekeeping genes (*Rn18s* and *Rpl13a)*. (E) HA levels in lung homogenates from uninfected or infected IL‐13 WT, HET, or KO mice at d7 p.i. as measured by ELISA. Values are normalized to lung weight. (F) Immunofluorescence staining of lung tissues to visualize bHABP (green) and cell nuclei (stained with 4′,6‐diamidino‐2‐phenylindole (DAPI), blue) in infected WT and KO mice at d7 p.i. White letters indicate regions of the lung; airways (A), vessels (V), or alveolar epithelium (*). Images are representative of *n* = 5 (WT) or *n* = 7 (KO) mice per group. Scale bar = 100 μm. (G) Quantification of HA staining intensity in alveolar epithelium at d7 in WT, HET, and KO mice. (H) Example of H&E‐stained lung sections from uninfected or IL‐13 WT or KO mice at d7 p.i. Images are representative or *n* = 5 (WT) or *n* = 7 (KO) per group. Scale bar: 200 μm (I) Quantification of lung damage in H&E sections by mean linear intercept analysis. In (A−E, G, and I), datapoints depict individual mice and graphs show mean ± SD. Comparisons between groups were determined by ANOVA with Tukey's multiple comparison test. Non‐normally distributed data were log‐transformed. *p < 0.05, **p < 0.01, ***p < 0.001, ****p < 0.0001.

Our data show that HA accumulates and a HC·HA matrix forms in the lung during Nb infection, coinciding with the induction of a type 2 immune response (Figures 1 and 3). As we have previously described IL‐13 as a regulator of pulmonary HA in other models [4, 6], we investigated HA matrix formation in IL‐13‐deficient mice infected with Nb. Consistent with previous observations [54], a reduced type 2 immune response, including decreased lung eosinophils at d7 p.i., was observed in IL‐13^*−/−*^ (KO) mice compared to infected IL‐13^*+/+*^ (WT) controls (Figure 4A). As expected, IL‐13 levels in the lung exhibited a gene‐dose effect with an increase upon infection in WT mice, which was reduced in HET mice and absent in the KO mice (Figure 4B). IL‐4 (similar to IL‐13 and which also signals through the IL‐4Rα) was increased in all infected mice (WT, HET, and KO) relative to uninfected animals but levels of IL‐4 in the lungs of IL‐13‐deficient mice were not significantly different to WT infected mice (Figure 4C). These findings were consistent with previous observations that other type 2 cytokines do not compensate for IL‐13 deficiency in this model [54]. Whole lung expression of the type 2 effector molecule *Retnla* was also significantly decreased in infected IL‐13 KO compared to infected WT mice (Figure 4D).

Having confirmed expected changes in the immune response in mice deficient in IL‐13, we next assessed the lung HA matrix. At d7 p.i., the infection‐induced accumulation of HA in lung homogenate was significantly reduced in IL‐13 KO mice (compared to infected WT littermate controls) (Figure 4E). A reduction in IL‐13 levels by half in IL‐13 HET mice was enough to reduce HA in lung homogenate, although this was not significant (Figure 4E). Given that we found that HA was increased in the alveolar parenchyma following infection in WT mice (Figure 1J), we next assessed HA localization in tissues sections (Figure 4F). As expected, HA was increased in the alveolar epithelium of IL‐13 WT‐infected mice but reduced in tissues of infected IL‐13 KO mice (Figure 4G). While IL‐13 KO had less HA in response to infection, the amount of alveolar epithelium damage (defined by mean linear intercept analysis) was increased in KO mice (Figure 4H). Together our findings are consistent with previous observations that IL‐13 signaling limits damage after Nb infection [54] in line with the established roles of IL‐13‐induced signaling molecules like RELMα in tissue repair [31, 35].

## 4 Discussion

Accumulation of HA in the lung has been described following viral infection, allergen challenge, and chemical damage [1, 2, 3, 5, 8, 9]. Here, we show that HA is also increased during injury induced by the lung‐migrating nematode Nb. Host immunity to infection with Nb has been thoroughly characterized [44, 49, 55, 56, 57] and includes eosinophilia, which peaks at d7 p.i., well after the parasite has exited the lungs. Timing of the type 2 immune response coincided with IL‐13‐dependent HA accumulation. This increase in HA was then resolved (by d15 p.i.), which is consistent with a time when Nb‐infected lungs undergo a degree of repair, as well as the known role of IL‐13 in tissue repair [38, 53].

IL‐13 signaling is essential for eosinophil recruitment in the lung following Nb infection [54]. Here, we observed during Nb infection that the peak HA response coincides with the peak of eosinophilia in the tissue. Correlation between HA deposition and eosinophilia has also been reported in models of asthma and allergic airway pathology [58, 59]. Indeed, HA has been shown to be important in eosinophil differentiation and proliferation [60] as well as eosinophil‐dependent TGF‐β signaling that contributes to (pathogenic) remodeling in asthma [58, 61]. While HA–eosinophil interactions in asthma likely promote aberrant tissue repair, it would be interesting to determine their contribution during Nb infection. IL‐13 signaling is also critical for the release of type 2 effector molecules, such as RELMα, which are associated with tissue repair [35, 62]. The effects of RELMα signaling have been investigated in relation to the collagen ECM, but not in relation to HA, despite both HA and collagen being essential for tissue repair through the formation of provisional matrices [63]. Here, we highlight that IL‐13‐deficient mice have reduced RELMα, reduced HA, and increased tissue damage. Therefore, understanding IL‐13‐RELMα‐HA signaling will provide important insight into pathways of effective versus ineffective tissue repair.

IL‐13 signals through the type II receptor, consisting of IL‐4Rα (shared with the IL‐4/type I receptor) and IL‐13Rα1 subunits [39, 64]. The type II receptor is expressed on immune cells but is also highly expressed on structural and stromal cells of the lung. Often, IL‐13 signaling is associated with pathological fibrotic wound responses in the lung [65, 66, 67]. For example, IL‐13 blockade has been shown to be beneficial in patients with idiopathic pulmonary fibrosis [68] and COPD [69]. However, in bleomycin‐induced lung injury, signaling via IL‐13Rα1 was shown to have protective effects. IL‐13Rα1 KO mice have reduced expression of wound repair genes (including *Retnla*) and increased collagen production, which results in more severe bleomycin‐induced damage [70]. During Nb infection, we observed that HA accumulation was localized to the alveolar epithelium, the region of the lung damaged during infection when parasites break through capillary beds [38, 54]. While our experiments have not identified the specific cell types involved in HA production, evidence from the literature would suggest that lung epithelial cells may be an important population [52]. Furthermore, such epithelial cells are capable of responding to IL‐13 (see *Tabula Muris* [71]). Adding to this, high‐molecular‐weight HA (HMW‐HA) is associated with epithelial survival post injury in the lung [72] and type II alveolar epithelial cells lacking HA have reduced renewal capabilities [73], a feature which is essential for healthy lung repair [74, 75, 76, 77]. The contributions of other cell types to the resulting HA matrix, such as fibroblasts [78, 79] or immune cells [52], also warrant investigation. Consistent with our previous findings [54], a lack of IL‐13 enhanced lung injury following Nb infection. Indeed, during infection, IL‐13 has multiple host‐protective roles, including parasite expulsion and limiting bleeding [45, 54]. Thus, in the context of Nb, we hypothesize that the repair‐promoting effects of both IL‐13 and HA are important.

During Nb infection, our data suggest that increased expression of *Has2* contributes to pulmonary HA accumulation. Interestingly, we found that *Has1* was suppressed during the peak HA response, which may indicate that *Has* isoforms in the lung are modulated by different aspects of the tissue response to Nb. Differential regulation of *Has* isoforms has been described in other lung challenge models, such as influenza, where *Has1* is induced at d3 p.i. coinciding with the innate response to infection, while *Has2* is increased at d6 p.i. once an adaptive response has begun [1, 2]. Previous in vitro studies have suggested that different mammalian Has isoforms may contribute to different sizes of HA chains [80, 81], which may influence the “flavor” of HA matrix produced in response to challenge. Concurrently, our data show that the peak type 2 response during Nb infection is also associated with suppression of *Hyal1*, *Hyal2,* and *Tmem2*. This could indicate an attempt to limit HA fragmentation (which can also be mediated by reactive oxygen species [82, 83, 84]) within the tissue during the peak of inflammation. Beneficial effects of exogenous high‐molecular‐weight HA have been demonstrated in numerous lung models, including asthma [85, 86] and sepsis [15, 87]. Low‐molecular‐weight HA fragments however have been associated with endothelial barrier dysfunction in COVID‐19 [88]. While we have not assessed the molecular weight of HA from lung tissue or BALF during Nb infection, it would be interesting to determine how the size profile compares to HA in the lung during other challenges [89].

Early during Nb infection, the parasite migrates through the lungs from the bloodstream [38], leading to innate neutrophilic responses that culminate in tissue damage [47, 90]. This timepoint corresponded to the peak of *Tnfaip6* (Tsg6) induction during Nb infection. This is interesting given that (human) TSG‐6 has been shown to inhibit neutrophil migration [91, 92]. Similarly, in other lung challenge models, such as LPS or bleomycin‐induced injury, Tsg6 is modulated early in response to damage [93]. Increased Tsg6 expression is consistent with the formation of a HC·HA matrix during Nb infection, given that Tsg6 is currently the only known transferase for this process [23, 25, 26]. HC·HA was most evident by d7 p.i., suggesting that this matrix takes time to form at a level detectable by our western blot analysis. Interestingly, no HC·HA matrix was detected at d15 or d30 indicating that this matrix is removed or depleted as part of the lung response to Nb infection and may represent a key determinant of appropriate repair. Similarly, we observed that HC·HA matrices colocalized to regions of HA accumulation and epithelial damage. During influenza infection, HC·HA complexes have been detected at d6 or d8 p.i [1, 2], with elevated HA levels persistent in the lungs after resolution of infection (measured up to d42 p.i. in BALF [1]). How long the HC·HA matrix persists in the lung tissue post flu infection has not been investigated, although rapid clearance (within 2 days) of HC·HA has been shown in an acute model of injury using LPS [94]. Here, we show that during Nb challenge, a HC·HA matrix is formed but then resolved (along with increased HA levels). Determining the specific composition of HCs within HC·HA matrices will be important to fully appreciate their roles in lung pathology and tissue repair. Aside from HC‐modification, interaction of HA with other HABPs, (e.g., versican, see [30]) is likely critical to the role of the HA matrix that forms p.i. Interestingly, IL‐13 has been shown to increase expression of versican in stimulated human asthmatic fibrocytes [95]. How HA matrices are formed and composed, with regard to HCs, HABPs, and accessory proteins (e.g. pentraxin‐3 [96]), along with how such matrices are removed from the tissue, particularly in response to different lung challenges, remain important questions for the field.

## 5 Conclusion

Data presented here provide further evidence that IL‐13 is a regulator of the HA matrix during lung injury. Understanding what factors drive HA in response to lung challenge is essential to better appreciate when and how HA matrices contribute to or impede lung repair. Further studies using the Nb model of immune‐mediated lung injury and repair will help reveal how type 2‐mediated HA matrices regulate appropriate tissue repair.

## Author Contributions

Conceptualization: Rebecca J. Dodd, Tara E. Sutherland, Judith E. Allen. Formal analysis: Rebecca J. Dodd, Dora Moffatt, Monika Vachiteva. Investigation: Rebecca J. Dodd, Dora Moffatt, Monika Vachiteva, James E. Parkinson. Resources: Anthony J. Day, Brian H. K. Chan. Writing original draft: Rebecca J. Dodd. Writing review and editing: Rebecca J. Dodd, Dora Moffatt, Monika Vachiteva, James E. Parkinson, Brian H. K. Chan, Anthony J. Day, Judith E. Allen, Tara E. Sutherland. Supervision: Tara E. Sutherland, Judith E. Allen, Anthony J. Day. Funding acquisition: Tara E. Sutherland, Judith E. Allen, Anthony J. Day.

## Conflicts of Interest

A.J.D. is a founder of Link Biologics Ltd. which is developing a TSG‐6‐based biological drug (unrelated to this study), and A.J.D. and R.J.D. are shareholders in the company. The remaining authors declare no conflicts of interest.

## Supporting information

Supporting Information.

Supporting Information.

Supporting Information.

## Data Availability

All relevant data are included within the manuscript and Supporting Information Materials.
