# Supplementary material for: Injury From Nematode Lung Migration Induces an IL‐13‐Dependent Hyaluronan Matrix
Source: Proteoglycan Res. 2024 Nov 25;2(4):e70012. doi: 10.1002/pgr2.70012 (PMC11589410; doi:10.1002/pgr2.70012)
Supplement: Supplementary file 1 — Supporting Information. [file PGR2-2-e70012-s001.pdf]

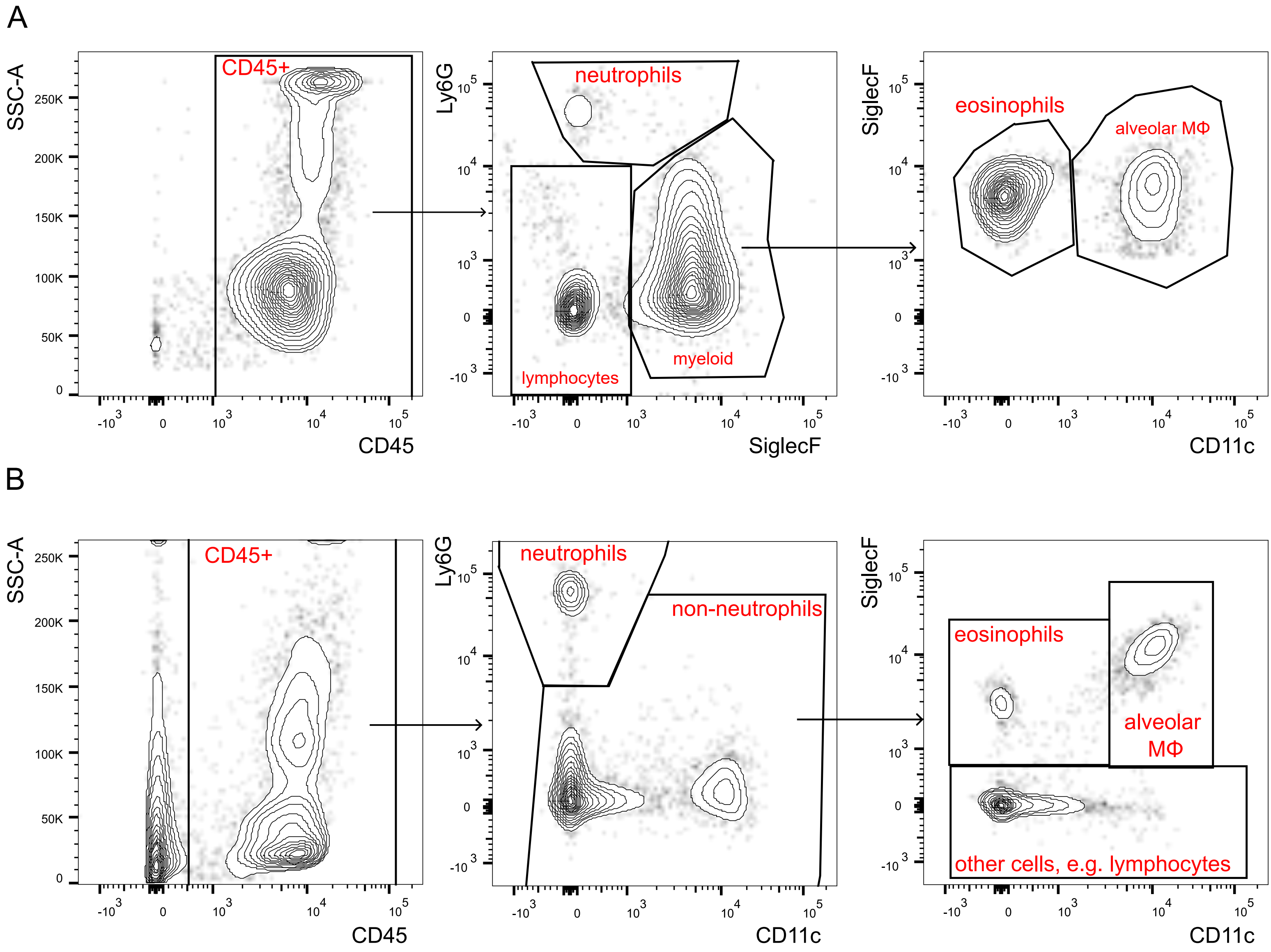

Supplementary Figure 1: Flow cytometry gating strategy to identify immune cell populations from A) BAL or B) lung. Details of antibodies used for FACS staining are given in Supplementary Table 1.
