# Supplementary material for: Injury From Nematode Lung Migration Induces an IL‐13‐Dependent Hyaluronan Matrix
Source: Proteoglycan Res. 2024 Nov 25;2(4):e70012. doi: 10.1002/pgr2.70012 (PMC11589410; doi:10.1002/pgr2.70012)
Supplement: Supplementary file 2 — Supporting Information. [file PGR2-2-e70012-s003.pdf]

Supplemental Figure 2

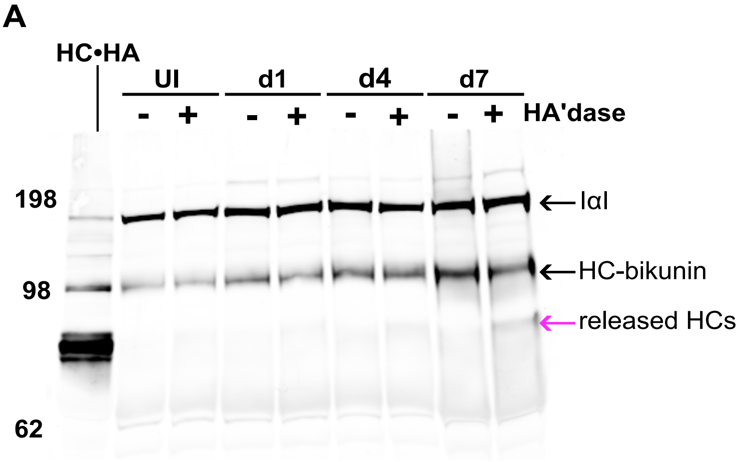

Supplementary Figure 2: Additional western blots of lung homogenates treated with and without hyaluronidase treatment to release HCs from HC•HA matrices present in tissue. **A**) Samples from d1, d4 and d7 p.i. compared to an uninfected control (UI) or **B**) samples from d7, d15 or d30 p.i. compared to UI control. Blots were probed with anti-**Iαl**.antibody and released HC bands were compared to those from synthetic in vitro HC•HA controls. d7 samples in A) and B) are from different mice from 2 seperate infection experiments. Blots are representative of at least three repeats with samples from different mice across 2 independent experiments.
