# Supplementary material for: Injury From Nematode Lung Migration Induces an IL‐13‐Dependent Hyaluronan Matrix
Source: Proteoglycan Res. 2024 Nov 25;2(4):e70012. doi: 10.1002/pgr2.70012 (PMC11589410; doi:10.1002/pgr2.70012)
Supplement: Supplementary file 3 — Supporting Information. [file PGR2-2-e70012-s002.docx]

**Supplemental Tables**

**Supplementary Table 1. Antibodies for flow cytometry staining of BAL and lung cells**

| **Antibody** | **Clone** | **Fluorophore** | **Dilution** | **Supplier** |
| --- | --- | --- | --- | --- |
| CD11b | M1/70 | BV605 or BV711 | 1:400 | BioLegend |
| CD11c | N418 | BV605 | 1:400 | BioLegend |
| CD4 | RM4-5 | BV650 | 1:400 | BioLegend |
| CD8 | 53-6.7 | BV711 | 1:400 | BioLegend |
| CD45 | 30-F11 | BV785 or PerCPCy5.5 | 1:400 | BioLegend |
| CD24 | M1/69 | F488 | 1:400 | eBioscience |
| CD44 | IM7 | PerCP-Cy5.5 | 1:400 | BioLegend |
| TCRγδ | GL3 | PE | 1:400 | BioLegend |
| CD3e | 145-2C11 | PeCy5 | 1:400 | BioLegend |
| CD90.2 | S3-2.1 | AF700 | 1:400 | BioLegend |
| TCRβ | H57-597 | AF780 | 1:400 | eBioscience |
| Ly6C | Hk1.4 | BV510 | 1:400 | BioLegend |
| CD64 | X54-5/7.1 | PE | 1:100 | BioLegend |
| SiglecF | E50-2440 | PeTxRed | 1:400 | BD Biosciences |
| CD19 | 6D5 | PeCy5 | 1:400 | BioLegend |
| Ly6G | 1A8 | PeCy7 | 1:400 | BioLegend |
| MerTK | 2b10c42 | APC | 1:100 | BioLegend |
| MHCII | M5/114.15.2 | AF780 | 1:1000 | BioLegend |

**Supplementary Table 2. Primers for qPCR analysis**

| **Gene** | **Forward primer sequence** | **Reverse primer sequence** |
| --- | --- | --- |
| *Rn18s* | GTAACCCGTTGAACCCCATT | CCATCCAATCGGTAGTAGCG |
| *Rpl13a* | CATGAGGTCGGGTGGAAGTA | GCCTGTTTCCGTAACCTCAA |
| *Has1* | CCACAGCCATGAGACAGGA | CCAAGGCCAGGAGTCCAT |
| *Has2* | TGAGTACAAAGAGGTTCGTT | ATTGTCAGGGTGTGTTTGTTT |
| *Has3* | CTACTTGTAGCTGCCCAGA | GAGTACAAAAAACAGCACC |
| *Hyal1* | CTTCTGCCCCTGGAGGAACT | GTGTGGAATCCATGTATGCT |
| *Hyal2* | CGAGGCATCACGGGACTGA | GCTGAGTTAGGTAATTCTTG |
| *Cemip* | CACCTAGGAGATGGTCTGGATA | GCACCCTCACTTACAGGAATAA |
| *Tmem2* | CTGCTCATTGTCCTACCAGAAA | CTCCATGATTCCACCCTAAGAAG |
| *Tnfaip6* | CCACGGCTTTGTAGGAAGAT | ATGCAGGATCCACTGTGACG |
| *Retnla* | TATGAACAGATGGGCCTCCT | GGCAGTTGCAAGTATCTCCAC |
| *Areg* | TAGCTGAGGACAATGCAGGGTA | AACTGGGCATCTGGAACCAT |

**Supplementary Table 3. Antibodies for immunofluorescence staining**

| **Antibody/binding protein** | **Dilution** | **Working concentration** | **Supplier** |
| --- | --- | --- | --- |
| biotinylated HA-binding protein (bHABP) | 1:200 | 2.5 μg/mL | Merck (385911) |
| rabbit polyclonal anti IαI | 1:5000 | - | Dako (A0301) |
| Streptavidin-557 | 1:200 | 50 μg/mL | R&DSystems (NL557) |
| Donkey anti-rabbit IgG-637 | 1:200 | - | R&DSystems (NL005) |
